# Toward clarifying ASAM’s inpatient and residential benzodiazepine tapering recommendations

**DOI:** 10.3389/fpsyt.2026.1857354

**Published:** 2026-05-26

**Authors:** Christopher K. Blazes, Stephen Leung, Doryn Davis Chervin, Bernard Silvernail

**Affiliations:** 1 Department of Psychiatry, Oregon Health and Science University, Portland, OR, United States; 2 Department of Psychiatry, University of California San Francisco, San Francisco, United States; 3 Alliance for Benzodiazepine Best Practices, Cherry Hill, NJ, United States; 4 Alliance for Benzodiazepine Best Practices, Portland, OR, United States

**Keywords:** ASAM, benzodiazepine, deprescribing, inpatient, residential, tapering, withdrawal

## 1 Introduction

The 2025 ASAM Joint Clinical Practice Guidelines on Benzodiazepine Tapering: Considerations When Risks Outweigh Benefits represent an important attempt to address the lack of evidence-based strategies for safely deprescribing benzodiazepines (1). While the authors acknowledge that most of the Guidelines rely on clinical consensus rather than empirical data, their publication is a meaningful step toward supporting clinicians and patients facing the complexities of benzodiazepine dependence and withdrawal.

Overall, we support the majority of the 42 recommendations. However, several recommendations involving inpatient and residential care require clarification to prevent misinterpretation and potential harm. Benzodiazepine tapering is a challenging clinical process with significant variability in patient needs (2), and the current phrasing may lead providers to over-refer—or underprepare for—the higher levels of care that they may have to provide.

## 2 Topics needing clarification

### 2.1 The role of inpatient care: indications and common misinterpretations

The Guidelines recommend inpatient admission when there is imminent risk of serious harm related to continued benzodiazepine use or when tapering is anticipated to be too complex or unsafe in an outpatient setting. Examples include acute suicidality, high-risk polypharmacy, overdose risk, or severe psychological dependence.

While inpatient care is sometimes appropriate, the summary section of the Guidelines does not highlight an essential clarification found in the full text:

“*Benzodiazepine tapering may need to occur across multiple settings, with patients beginning in inpatient care for stabilization but completing their taper in outpatient practice*”

This nuance is critical. Referring providers should not expect that inpatient units will fully discontinue benzodiazepines before discharge, nor should they relinquish ongoing responsibility for the taper. Without explicit emphasis, readers may incorrectly assume that inpatient settings are intended to accomplish complete discontinuation. The guideline should note that, while critical cases may receive hospital-level interventions for short durations to manage high-risk complications, this will usually need to be followed by outpatient treatment to complete the taper. Additionally, the guideline should note that, in order to reduce the high relapse rate, the patient will need to be monitored regularly post-discontinuation to manage ongoing withdrawal symptoms.

### 2.2 Insurance, access barriers, and practical limitations

The Guidelines offer little practical guidance on when and how to refer to inpatient or residential care. In reality, insurance authorization for these settings is inconsistent and often time-limited—sometimes to only a few days—requiring repeated reviews and re-authorizations. Residential care may not be covered at all without a formal substance use disorder diagnosis. Many patients who experience challenges during benzodiazepine tapering do not meet the criteria for a benzodiazepine use disorder (BUD) but may not qualify for insurance reimbursement without a BUD diagnosis.

These systemic barriers can result in incorrect BUD diagnoses or unsafe or incomplete tapers, emphasizing the need for clearer ASAM recommendations on referral pathways and realistic expectations for care transitions.

### 2.3 The ambiguity of “severe or complicated withdrawal”

Multiple recommendations hinge on whether a patient is experiencing, or is anticipated to experience, “severe or complicated withdrawal.” However, this term is not defined in the Guidelines.

Without a clear definition, clinicians may conclude that difficult cases should default to inpatient management or that inpatient/residential centers are inherently better equipped for challenging tapers. This risks oversimplifying clinical judgment.

Outpatient tapers can still be appropriate—even for complex cases—when carefully structured. Conversely, some patients require inpatient stabilization not because of withdrawal severity but because the taper is clinically necessary yet not feasible in outpatient settings (e.g., patients unwilling to taper despite risk, significant comorbidities, or behavioral factors especially those with underlying use disorders). This practical dilemma is not adequately acknowledged in the Guidelines.

### 2.4 Phenobarbital tapers: limited guidance and real-world challenges

The Guidelines allow for phenobarbital-based inpatient tapers, especially in urgent safety situations or for patients preferring rapid discontinuation (3–5). However, they provide no criteria to guide clinicians on which patients should receive phenobarbital versus a standard benzodiazepine taper.

Although phenobarbital detoxification is widely used in addiction treatment settings, evidence remains limited, and complications—including benzodiazepine-induced neurological dysfunction (BIND)—are documented (6). Clinicians often struggle to identify facilities experienced in phenobarbital tapers, further limiting its real-world applicability.

Given these gaps, ASAM should better delineate appropriate patient characteristics, risks, and indications for phenobarbital use for benzodiazepine discontinuation.

### 2.5 Recognizing protracted withdrawal and BIND

The Guidelines acknowledge the possibility of protracted withdrawal lasting months or years, but current recommendations underestimate how frequently clinicians fail to recognize ongoing symptoms. These long-term effects—including BIND—may persist after inpatient detoxification, making it difficult to distinguish withdrawal from underlying disorders without careful follow-up (7).

There should be no expectation that patients will complete their taper in an inpatient setting (other than a phenobarbital taper). Patients who complete an inpatient taper but receive inadequate outpatient monitoring risk subsequent misdiagnosis, inappropriate medication changes, or repeated cycles of withdrawal instability.

## 3 Discussion: specific areas where ASAM should revise and clarify

To ensure patient safety and prevent misinterpretation, we recommend that ASAM revise the Guidelines to:

1. Clearly state that inpatient care is primarily for stabilization, and most patients will still require long-term outpatient tapering afterward.
2. Define “severe or complicated withdrawal” with specific clinical criteria.
3. Emphasize in the Summary of Recommendations that outpatient monitoring and/or continuation of ongoing tapering is often necessary even after inpatient stays.
4. Specify when phenobarbital tapers are preferred over benzodiazepine tapers for patients with substance use disorders or those seeking rapid discontinuation.
5. Acknowledge the limited availability of facilities skilled in phenobarbital-based benzodiazepine detoxification.
6. Encourage providers individualize treatment throughout all the twists and turns of the deprescribing process.

These improvements would help avoid inappropriate and/or ineffective referrals to higher levels of care and support clinicians in making safer, more consistent treatment decisions.
